# Antigenotoxic Effect of Curcumin and Carvacrol against Parathion Induced DNA Damage in Cultured Human Peripheral Blood Lymphocytes and Its Relation to GSTM1 and GSTT1 Polymorphism

**DOI:** 10.1155/2014/404236

**Published:** 2014-09-25

**Authors:** Neeraj Kumar, Anita Yadav, Sachin Gulati, Kanupriya, Neeraj Aggarwal, Ranjan Gupta

**Affiliations:** ^1^Department of Biotechnology, Kurukshetra University Kurukshetra, Haryana 136119, India; ^2^Department of Microbiology, Kurukshetra University Kurukshetra, Haryana 136119, India; ^3^Department of Biochemistry, Kurukshetra University Kurukshetra, Haryana 136119, India

## Abstract

In recent years, the use of organophosphorus pesticides has been extensively increased and these compounds signify a major class of agricultural pesticides today. We studied antigenotoxic potential of curcumin and carvacrol against the parathion induced DNA damage in cultured peripheral blood lymphocytes using sister chromatid exchanges as a biomarker of genotoxicity. Heparinised fresh blood from healthy individuals was treated with 2.5 *μ*g/mL concentration of parathion in presence of curcumin and carvacrol in order to observe the antigenotoxic potential of both curcumin and carvacrol. Significant reduction (*P* < 0.05) was observed in the frequencies of SCEs in presence of 10 *μ*g/mL and 15 *μ*g/mL concentrations of curcumin as compared to parathion exposed sample. Similarly carvacrol had significant (*P* < 0.05) antigenotoxic effect at the concentrations of 2.5 *μ*g/mL and 5.0 *μ*g/mL against the parathion. We also studied the effect of GSTT1 and GSTM1 on genotoxicity of parathion and antigenotoxic potential of curcumin and carvacrol. We did not observe any significant effect (*P* > 0.05) of GSTT1 and GSTM1 polymorphism on genotoxicity of parathion and antigenotoxic potential of curcumin and carvacrol.

## 1. Introduction

Pesticides are ubiquitous contaminants of our environment and are extensively used all over the world. They include a vast diversity of substances used to kill, destroy, or repel unwanted living organisms. In recent years, public concerns have been raised about the potential risks of pesticides to human health. In our study we investigated the protective role of curcumin and carvacrol against the DNA damage caused by parathion in cultured human peripheral blood using sister chromatid exchanges (SCEs) as a biomarker of DNA damage. Parathion is one of the most commonly used organophosphorus insecticides. It has been used for bean, corn, sorghum, and tobacco crops to eliminate the green flies, harvest bugs, and other insects. It hinders the enzyme cholinesterase, responsible for hydrolyzing the acetylcholine to choline at the axon-terminals [1]. In absence of active cholinesterase, acetylcholine is accumulated and blocks the normal transmission of nerve impulses at the synapse which ultimately leads to loss of muscular coordination, convulsion, and death [2, 3]. Parathion has been reported to induce testicular damage in male rats [4].

Nutraceuticals are defined as any natural bioactive, chemical compounds that give medical or health benefits, including the prevention and treatment of disease. Curcumin (diferuloylmethane), a polyphenol, is the active ingredient of the dietary spice turmeric (*Curcuma longa*) and has been consumed for medicinal purposes for thousands of years [5], which has persuasive anticancer properties as demonstrated in a plethora of human cancer cell lines/animal carcinogenesis model. It acts as a free radical scavenger and antioxidant [6] inhibiting lipid peroxidation and oxidative DNA damage [7]. Curcumin can significantly decrease the frequencies of micronucleated polychromatic erythrocytes in mice exposed to gamma-radiation [8].

Carvacrol (5-isopropyl-2-methylphenol), used in this study, is monoterpenic phenol which is present in many essential oils of the family Labiatae including* Origanum*,* Satureja*,* Thymbra*,* Thymus*, and* Coridothymus *species and is used in our daily life such as cosmetic ingredient, safe food additive in baked goods, sweets, beverages, and chewing gum. It has been shown to exhibit a range of biological activities like antibacterial [9], antifungal, insecticidal [10], analgesic [11], and antioxidant [12] activities. In the recent past, it is revealed that carvacrol has antiproliferative properties on non-small cell lung cancer cells A549, chronic myeloid leukemia cells K562, Hep-2 cells, murine B16 melanoma cells, and human metastatic breast cancer cells, MDA-MB231 [13–17].

Molecular epidemiological studies have revealed that individual's susceptibility to mutagen depends on both genetic and environmental factors. The hereditary differences in the detoxification/activation of carcinogens play a crucial role in host vulnerability. Glutathione S-transferases (GSTs) are one among the most frequently studied polymorphisms regarding the metabolism of xenobiotics. The GSTM1 and GSTT1 are members of the glutathione S-transferase multigene family and are mostly concerned with the detoxification of a broad range of environmental carcinogens, endogenously produced reactive oxygen species (ROS), and lipid peroxidation products, yielding excretable hydrophilic metabolites [18]. There are only a few studies showing the effect of genetic polymorphism of GSTM1 and GSTT1 on genotoxicity of parathion. We studied the effect of GSTM1 and GSTT1 polymorphism on genotoxicity of parathion/antigenotoxicity of curcumin and carvacrol as measured by SCE frequency in cultured peripheral blood lymphocytes under* in vitro* conditions.

## 2. Materials and Methods

### 2.1. Sample Collection

5 mL venous blood was taken from healthy individuals in two separate vacutainer tubes containing sodium heparin and dipotassium ethylenediamine tetraacetic acid (EDTA) for lymphocyte culture set-up and DNA extraction, respectively. The protocol was duly approved by Human Ethical Committee of Kurukshetra University.

### 2.2. Human Lymphocyte Culture

Short term peripheral blood lymphocyte (PBL) cultures were set up using earlier studied technique of Moorhead et al. [19] with minor modifications. Culture was set up in duplicate by adding (0.4 mL) whole heparinized blood into 5 mL of RPMI 1640 culture medium (Himedia) containing L-glutamine (1%), fetal calf serum (20%) (Himedia), penicillin (100 UI/mL) and streptomycin (100 *μ*g/mL) solution (Himedia), and phytohaemagglutinin (2%) (Bangalore genei). The cultures were incubated at 37°C and 5% CO_2_ for 72 hours.

### 2.3. Sister Chromatid Exchange (SCE)

For sister chromatid exchange (SCE) analysis, 5-bromo-2-deoxyuridine (Sigma) was added after 24 hours of incubation in final concentration of 10 *μ*g/mL of culture. Parathion (Sigma) was added at the beginning of culture in concentrations ranging from 0.5 to 5 *μ*g/mL. Maximum genotoxic dose of parathion, that is, 2.5 *μ*g/mL, was chosen to check the protective effect of curcumin and carvacrol (Sigma). To check antigenotoxic potential of curcumin and carvacrol against parathion, cultures were set up separately having various combinations of parathion and curcumin/carvacrol. In one set-up, heparinised fresh blood was treated with 2.5 *μ*g/mL concentration of parathion along with 10 and 15 *μ*g/mL concentrations of curcumin while in others 2.5 and 5.0 *μ*g/mL concentrations of carvacrol were added against 2.5 *μ*g/mL concentration of parathion. Combined effect of both curcumin and carvacrol was also checked against parathion. Blood was also treated with curcumin and carvacrol alone to check their genotoxic effects if any. Blood without any mutagen/curcumin and carvacrol acted as control while blood having dimethyl sulphoxide was taken as negative control. The cultures were then incubated for 72 hrs at 37°C and 5% CO_2_. Colchicine (Sigma) was added 45 minutes prior to the harvesting in final concentration of 0.2 *μ*g/mL. The cells were harvested by centrifugation and then treated with hypotonic solution (0.075 M KCl) and fixed in methanol : acetic acid (3 : 1). From a suspension of fixed cells, slides were prepared by the air drying method and stained with Hoechst 33258 (Sigma) and 4% Giemsa stain (Himedia) solution following the method of Perry and Wolff. For calculating the frequency of SCE per cell, 50 metaphase plates were analyzed.

### 2.4. GSTM1 and GSTT1 Genotyping

Genomic DNA was extracted from 200 *μ*L of whole blood by DNA extraction kit (Bangalore genei). Multiplex PCR was used to detect the presence or absence of GSTM1 and GSTT1 genes. For the purpose of internal control, a part of exon 7 of the constitutional gene CYP1A1 was also coamplified. The amplification reaction was carried out in a 25 *μ*L volume containing 50–100 ng of genomic DNA as a template, 20 pmol/*μ*L of each primer (GenXbio), 200 *μ*M of each dNTP (Bangalore genei), 1X PCR buffer with 15 mM/L MgCl_2_ (Bangalore genei), and 0.7 units of Taq polymerase (Bangalore genei). PCR was performed by using reaction mixture with primers GSTM1 (Fw 5′-GAACTCCCTGAAAAGCTAAAGC-3′ and Re 5′-GTTGGGCTCAAATATACGGTGG-3′); GSTT1 (Fw 5′-TTCCTTACTGGTCCTCACATCTC-3′, Re 5′-TCACGGGATCATGGCCAGCA-3′); and CYP1A1 (Fw 5′-GAACTGCCACTTCAGCTGTCT-3′ and Re 5′-CAGCTGCATTTGGAAGTGCTC-3′) yielding a 312-bp product. Initial denaturation was done at 94°C for 10 min. A total of 35 temperature cycles were used: 94°C at 1 min, 59°C for 30 sec, and 72°C for 1 min. The last elongation step was extended to 10 mins at 72°C. The PCR products were analyzed in 2% agarose gel.

### 2.5. Statistical Analysis

All treatments were performed in duplicate and results were expressed as means ± S.E. The Student's *t*-test was used for calculating the statistical significance using SPSS 16.0.

## 3. Results

### 3.1. SCE Results

**Figure 1:**
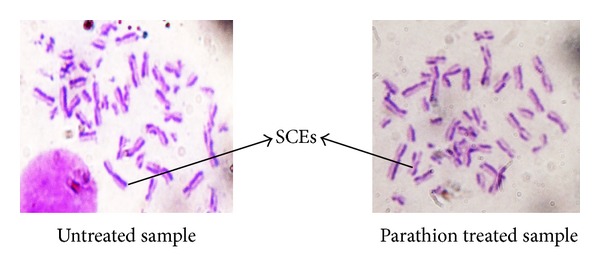
SCEs shown in untreated and parathion treated sample.

**Figure 2:**
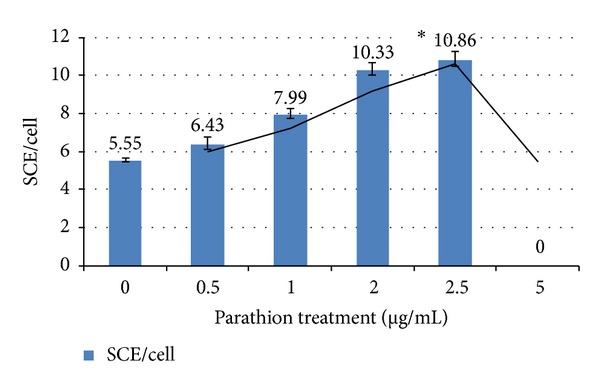
Induction of SCE in cultured lymphocytes by parathion. *P < 0.05 (significance as compared to untreated).

We studied the protective effects of curcumin and carvacrol against parathion induced genotoxicity in cultured peripheral blood lymphocytes. Sister chromatid exchanges (SCEs) were examined to assess the genotoxicity of parathion and antigenotoxic potential of curcumin and carvacrol. An increased frequency of SCEs was observed in parathion treated sample as compared to untreated sample (Figure 1). Parathion was observed alone for its mutagenicity by using various concentrations. Among different concentrations of parathion, 2.5 *μ*g/mL concentration had shown significant increase in SCE as compared to untreated sample (Table 1 and Figure 2) and this concentration was chosen further for analyzing antigenotoxic potential of curcumin and carvacrol.

**Figure 3:**
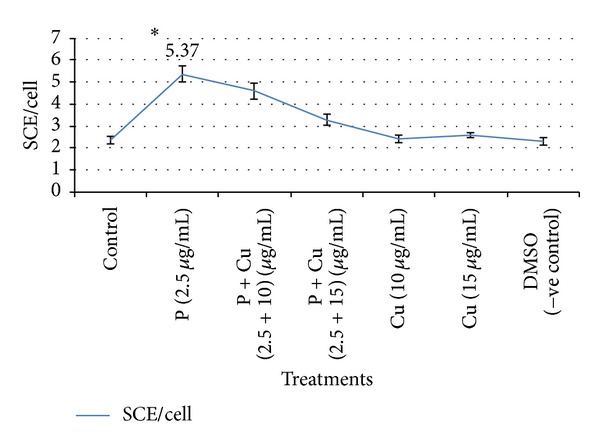
Reduction in SCE frequency by curcumin against parathion treated cultured human lymphocytes. *P < 0.05 (significance as compared to untreated); P: parathion, Cu: curcumin, and DMSO: dimethyl sulphoxide.

**Figure 4:**
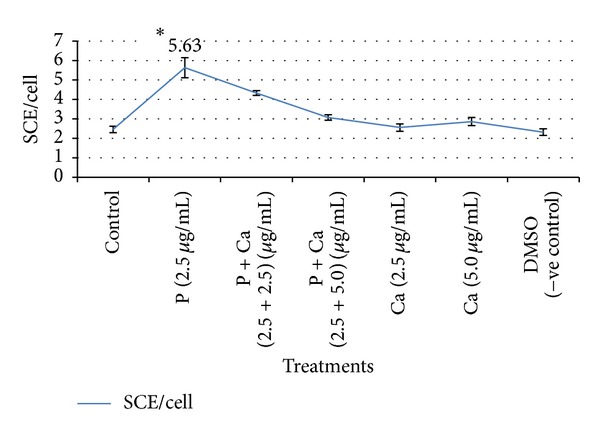
Ameliorative effect of carvacrol against parathion treated cultured human lymphocytes. *P < 0.05 (significance as compared to untreated), P: parathion, Ca: carvacrol, and DMSO: dimethyl sulphoxide.

**Figure 5:**
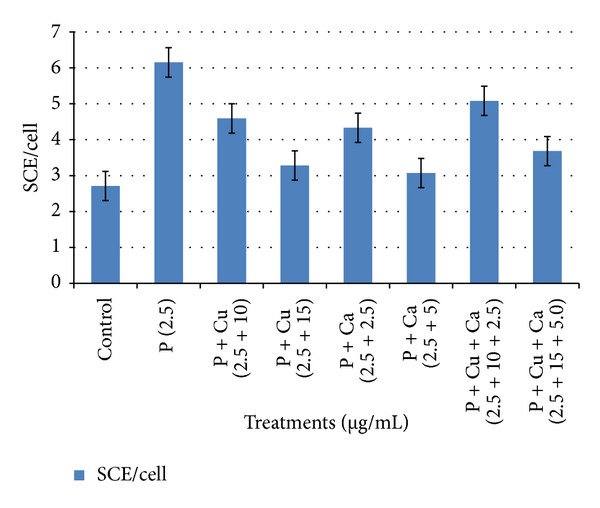
Simultaneous effects of curcumin and carvacrol in SCE assay in human lymphocytes against parathion. *P: parathion, Cu: curcumin, and Ca: carvacrol.

Antigenotoxic effect of curcumin and carvacrol was analysed by reduction in SCE frequency in presence of parathion. Curcumin at the concentrations of 10 and 15 *μ*g/mL had significantly reduced the SCE frequency as compared to parathion treatment (Table 2 and Figure 3). Similarly carvacrol had protective effect at the concentrations 2.5 and 5.0 *μ*g/mL (Table 3 and Figure 4). Both curcumin and carvacrol were also analysed for any genotoxic effect in absence of parathion. None of these were observed to be genotoxic. Combined effect of both curcumin and carvacrol was also studied (Table 4 and Figure 5). It was found that reduction in SCE frequencies was higher with combined treatment of curcumin and carvacrol as compared to their alone treatment. However this reduction was not found to be significant.

### 3.2. Effect of Genetic Polymorphism of GSTM1 and GSTT1 on Genotoxicity of Parathion and Antigenotoxicity of Curcumin and Carvacrol

Individuals respond differently to environmental chemicals due to differences in their genotypes. Multiplex PCR was used to detect the presence or absence of GSTM1 and GSTT1 genotypes. The occurrence of GSTM1 and GSTT1 genes was detected by the presence or absence of a band at 215 and 480 bp, respectively. We observed the effect of GSTM1 and GSTT1 polymorphism on genotoxicity of parathion and antigenotoxicity of curcumin and carvacrol. It was found that individuals which are nonnull for both GSTM1 and GSTT1 genes have less genotoxicity in presence of parathion as compared to those who either are null for both GSTM1 and GSTT1 genes or have either one of them (Table 5). However genotoxicity was not found to be significant. Similarly curcumin was found to be more antigenotoxic in individuals having GSTM1 and GSTT1 nonnull genotypes as compared to those having GSTM1 and GSTT1 null genotypes. However this antigenotoxic effect was not found to be significant.

Our study supports that curcumin and carvacrol have possible protective effects against the parathion while their combined treatment did not show any significant reduction in frequencies of SCEs as compared to their separate treatments. It was also observed that there is not any significant effect of GSTT1 and GSTM1 polymorphism on parathion induced genotoxicity and antigenotoxic effect of curcumin and carvacrol.

## 4. Discussion

In present study, we investigated the protective effect of curcumin and carvacrol and their combination against the genotoxic damage caused by parathion using sister chromatid exchange (SCE) as a biomarker of genotoxicity. Sister chromatid exchange (SCE) is a more sensitive indicator of genotoxic effects [20]. We observed the dose dependent increases in the frequency of SCEs by parathion at concentrations ranges from 0.5 to 5.0 *μ*g/mL with maximum damage at 2.5 *μ*g/mL. Parathion is reported to be genotoxic in our study as supported by the literature also. Sandra et al. [21] reported that human lymphocytes treated with 0 to 10 ppm concentration of parathion had significantly increased the SCE frequency in a dose dependent manner. However, at 13 ppm, cells failed to differentiate. Methyl parathion was also found genotoxic to human peripheral blood lymphocytes under* in vitro* conditions [22]. Rojas-García et al. [23] reported the dose dependent increase in micronucleus in peripheral human blood lymphocytes with paraoxon (an active metabolite of parathion). They treated the peripheral human blood lymphocytes with range of 1–25 *μ*M concentrations of paraoxon and found dose dependent DNA damage. Chen et al. [24] reported the increases in frequency of SCE in Chinese hamster V79 cell line treated with methyl parathion. They treated the cells with 10, 20, and 40 *μ*g/mL concentrations of methyl parathion at the time intervals of 28, 34, and 72 hours. They found that methyl parathion increases the SCE frequency in a dose dependent manner as the concentration increases from 10 to 40 *μ*g/mL. Maximum genotoxic damage was observed at 40 *μ*g/mL concentration of methyl parathion.

Nowadays, curcumin is appearing as a promising chemopreventive compound able to reverse, inhibit, or prevent the development of cancer by inhibiting specific molecular signaling pathways involved in carcinogenesis [25–28]. It is well known for its antioxidant property [29, 30]. Its free radical scavenging antioxidant property helps it in reducing genotoxic damage. Antimutagenic property of carvacrol has not been well studied yet [31]. Its antimutagenic effect may be due to its antioxidant nature [12]. Genotoxic potential of carvacrol was found to be very weak in both Ames and DNA-repair test [32].

In our study, we observed that 10 and 15 *μ*g/mL of curcumin have significantly reduced the genotoxic damage caused by parathion which supports its antigenotoxic property. We also observed that carvacrol had protective effect against parathion at the concentrations of 2.5 and 5.0 *μ*g/mL, supporting its antigenotoxic activities. Combination of curcumin and carvacrol was also analysed against the parathion. Curcumin and carvacrol in combination have reduced the frequencies of SCEs as compared to parathion but results were not significant in comparison to their separate treatments. As in our study, some other workers have also reported the antigenotoxic effects of curcumin and carvacrol. Under* in vitro* conditions, curcumin is reported to reduce the clastogenic effects of gamma-radiation in human lymphocytes culture. Lymphocytes pretreated with curcumin exposed to 1 and 2 Gy (SI unit of absorbed dose of radiations) of gamma-radiation resulted in decreased frequency of SCEs as compared to untreated lymphocytes [33]. Curcumin at the doses of 5, 10, and 15 *μ*M had shown the dose dependent decrease in SCEs/cell against 10 *μ*g/mL concentration of tinidazole [34]. Carvacrol was reported to inhibit the rate of SCE induced by mitomycin C [35]. Ultee et al. [36] reported that the antimutagenic activity of carvacrol in* B. cereus* IFR-NL94-25 against parathion might be due to alteration in membrane lipids and permeability of ion channels, thus inhibiting the uptake of parathion into the cells.

Individuals have different responses to environmental chemicals due to their different genotypes. In the present study, we studied the effect of GSTT1 and GSTM1 polymorphism on genotoxicity of parathion and the antigenotoxic effect of curcumin and carvacrol. We found no significant effect of GSTM1 and GSTT1 polymorphism on parathion induced genotoxicity and antigenotoxicity of curcumin and carvacrol under* in vitro* conditions. Similar to our findings Güven et al. [37] also reported that GSTM1 genotype did not influence the SCEs and CAs induced* in vitro* by BaP. Lack of the GSTM1 gene has no influence on induction of micronuclei by BaP in human lymphocyte cultures [38]. Kumar et al. [39] had reported no significant relationship between CYP1A1, GSTM1, GSTT1, and GSTP1 polymorphism and genotoxicity of trichloroethylene under both* in vivo* and* in vitro* conditions. To the best of our knowledge, there are no studies regarding the antigenotoxic effect of curcumin and carvacrol against parathion induced genotoxicity in human peripheral blood lymphocytes under* in vitro* conditions. Ours is the first report. Our findings suggest that curcumin and carvacrol may be administered in diet as a preventive measure against common genotoxicants and may further be used for the development of safer medication against impacts of carcinogens.

## Figures and Tables

**Table 1:** Frequency of SCE/cell in cultured human lymphocytes treated with parathion.

Parathion treatment (*µ*g/mL)	Metaphase scored	SCE/cell ± SE
Untreated	50	5.55 ± 0.10
0.5	50	6.43 ± 0.34
1	50	7.99 ± 0.25
2	50	10.33 ± 0.32
**2.5**	**50**	10.86 ± 0.40^a^
5.0	50	No differentiation

^a^ *P* < 0.05 (significance as compared to untreated).

**Table 2:** Antigenotoxic effect of curcumin against parathion treated cultured human lymphocytes.

Treatments	Concentrations used (*μ*g/mL + *μ*g/mL)	Metaphase scored	SCE/cell ± SE
Control	Untreated	50	2.33 ± 0.16
Parathion	2.5	50	5.37 ± 0.36^a^
Parathion + curcumin	2.5 + 10	50	4.59 ± 0.38^b^
Parathion + curcumin	2.5 + 15	50	3.28 ± 0.24^b^
Curcumin	10	50	2.41 ± 0.17^c^
Curcumin	15	50	2.57 ± 0.13^c^
DMSO (−ve control)	20	50	2.32 ± 0.17^d^

^a^ *P* < 0.05 (significance as compared to untreated), ^b^ *P* < 0.05 (significant as compared to parathion treatment), ^c^ *P* > 0.05 (nonsignificance as compared to untreated), and ^d^ *P* > 0.05 (nonsignificance as compared to untreated).

**Table 3:** Protective effect of carvacrol against parathion treated cultured human peripheral blood lymphocytes.

Treatments	Concentrations used (*μ*g/mL + *μ*g/mL)	Metaphase scored	SCE/cell ± SE
Control	Untreated	50	2.46 ± 0.16
Parathion	2.5	50	5.63 ± 0.52^a^
Parathion + carvacrol	2.5 + 2.5	50	4.33 ± 0.12^b^
Parathion + carvacrol	2.5 + 5.0	50	3.07 ± 0.14^b^
Carvacrol	2.5	50	2.56 ± 0.19^c^
Carvacrol	5.0	50	2.86 ± 0.21^c^
DMSO (−ve control)	20	50	2.32 ± 0.17^d^

^a^ *P* < 0.05 (significance as compared to untreated), ^b^ *P* < 0.05 (significant as compared to parathion treatment), ^c^ *P* > 0.05 (nonsignificance as compared to untreated), and ^d^ *P* > 0.05 (nonsignificance as compared to untreated).

**Table 4:** Combined effect of curcumin and carvacrol against parathion treated cultured human peripheral blood lymphocytes.

Treatments	Concentrations used (*μ*g/mL + *μ*g/mL)	Metaphase scored	SCE/cell ± SE
Control	Untreated	50	2.71 ± 0.22
Parathion	2.5	50	6.15 ± 0.65^a^
Parathion + curcumin	2.5 + 10	50	4.59 ± 0.38^b^
Parathion + curcumin	2.5 + 15	50	3.28 ± 0.24^b^
Parathion + carvacrol	2.5 + 2.5	50	4.33 ± 0.12^b^
Parathion + carvacrol	2.5 + 5.0	50	3.07 ± 0.14^b^
Parathion + curcumin + carvacrol	2.5 + 10 + 2.5	50	5.08 ± 0.41^c^
Parathion + curcumin + carvacrol	2.5 + 15 + 5.0	50	3.68 ± 0.31^c^

^a^ *P* < 0.05 (significance as compared to untreated).

^b^ *P* < 0.05 (significant as compared to parathion treatment).

^c^ *P* > 0.05 (nonsignificant as compared to curcumin and carvacrol alone treatment).

**Table 5:** Effect of GSTT1 and GSTM1 polymorphism on antigenotoxicity of curcumin and carvacrol against parathion induced genotoxicity.

Genotype	Parathion (2.5 *μ*g/mL)	Parathion (2.5 *μ*g/mL) + curcumin (10 *μ*g/mL)	Parathion (2.5 *μ*g/mL) + carvacrol (2.5 *μ*g/mL)
Relationship with GSTT1			
GSTT1 (nonnull)	5.90 ± 0.41	4.50 ± 0.35	4.26 ± 0.21
GSTT1 (null)	5.27 ± 0.38	3.85 ± 0.22^a^	4.22 ± 0.10^a^

Relationship with GSTM1			
GSTM1 (nonnull)	5.93 ± 0.85	4.36 ± 0.38	4.30 ± 0.15
GSTM1 (null)	6.00 ± 0.55	4.33 ± 0.66^b^	4.43 ± 0.31^b^

Relationship with both GSTT1 and GSTM1			
GSTT1 (nonnull), GSTM1 (nonnull)	4.80 ± 0.16	3.80 ± 0.17	4.20 ± 0.20
GSTT1 (null), GSTM1 (null)	5.45 ± 0.44	4.02 ± 0.0.34^c^	4.15 ± 0.25^c^
GSTT1 (nonnull), GSTM1 (null)	6.10 ± 0.40	4.35 ± 0.50	5.00 ± 0.17
GSTT1 (null), GSTM1 (nonnull)	6.50 ± 0.44	4.65 ± 0.45^d^	4.26 ± 0.12^d^

^a^ *P* > 0.05 (nonsignificance as compared to GSTT1 nonnull genotypes).

^b^ *P* > 0.05 (nonsignificance as compared to GSTM1 nonnull genotypes).

^c^ *P* > 0.05 (nonsignificance as compared to GSTT1 and GSTM1 nonnull genotypes).

^d^ *P* > 0.05 (nonsignificance as compared to GSTT1 nonnull and GSTM1 null genotypes).
